# Random forest of perfect trees: concept, performance, applications and perspectives

**DOI:** 10.1093/bioinformatics/btab074

**Published:** 2021-02-01

**Authors:** Jean-Michel Nguyen, Pascal Jézéquel, Pierre Gillois, Luisa Silva, Faouda Ben Azzouz, Sophie Lambert-Lacroix, Philippe Juin, Mario Campone, Aurélie Gaultier, Alexandre Moreau-Gaudry, Daniel Antonioli

**Affiliations:** Techniques de l’Ingénierie Médicale et de la Complexité - Informatique, Mathématiques, Applications (TIMC—IMAG) -UMR 5525, Université Grenoble Alpes—CNRS, France; CRCINA - INCIT Department - Team 2 - 8, quai Moncousu - BP 70721 - 44007 Nantes cedex 1 , France; Institut de Cancérologie de l’Ouest, Bd Jacques Monod, Unité de Bioinfomique, Saint Herblain Cedex, 44805, France; Techniques de l’Ingénierie Médicale et de la Complexité - Informatique, Mathématiques, Applications (TIMC—IMAG) -UMR 5525, Université Grenoble Alpes—CNRS, France; École Centrale de Nantes, High Performance Computing Institute, Nantes Cedex 3, 44321, France; Institut de Cancérologie de l’Ouest, Bd Jacques Monod, Unité de Bioinfomique, Saint Herblain Cedex, 44805, France; Département STID, IUT2 de Grenoble—Université Grenoble Alpes, St Martin d’Heres, 38400, France; CRCINA, INSERM, CNRS, Université de Nantes, Université d'Angers, Institut de Recherche en Santé-Université de Nantes, Nantes Cedex 1, 44007, France; Oncologie Médicale, Institut de Cancérologie de l’Ouest—René Gauducheau, Saint Herblain Cedex, 44805, France; Nantes Department of General Practice, 1 rue G. Veil, 44000, Nantes, France; Techniques de l’Ingénierie Médicale et de la Complexité - Informatique, Mathématiques, Applications (TIMC—IMAG) -UMR 5525, Université Grenoble Alpes—CNRS, France; Medical Informatics, Tournemire, Quartier Bellevue, France

## Abstract

**Motivation:**

The principle of Breiman's random forest (RF) is to build and assemble complementary classification trees in a way that maximizes their variability. We propose a new type of random forest that disobeys Breiman’s principles and involves building trees with no classification errors in very large quantities. We used a new type of decision tree that uses a neuron at each node as well as an in-innovative half Christmas tree structure. With these new RFs, we developed a score, based on a family of ten new statistical information criteria, called Nguyen information criteria (NICs), to evaluate the predictive qualities of features in three dimensions.

**Results:**

The first NIC allowed the Akaike information criterion to be minimized more quickly than data obtained with the Gini index when the features were introduced in a logistic regression model. The selected features based on the NICScore showed a slight advantage compared to the support vector machines—recursive feature elimination (SVM-RFE) method. We demonstrate that the inclusion of artificial neurons in tree nodes allows a large number of classifiers in the same node to be taken into account simultaneously and results in perfect trees without classification errors.

**Availability and implementation:**

The methods used to build the perfect trees in this article were implemented in the ‘ROP’ R package, archived at https://cran.r-project.org/web/packages/ROP/index.html.

**Supplementary information:**

Supplementary data are available at *Bioinformatics* online.

## 1 Introduction 

The principle of Breiman's random forest (RF) is to build and assemble complementary classification trees in a way that maximizes their variability (Breiman, 2001). The nodes of these trees contain only one variable. As a result, the predictive information of one variable does not take into account other variables, and its effects cannot be adjusted by those of other variables. In biology, events are multifactorial, and the effect of each variable is modulated by thousands of other variables. In addition, in classification and regression tree (CART) modeling, a hierarchical structure between variables that is defined in a conditional and ordered manner is assumed. At each step, only one variable is involved. This algorithmic structure is inadequate for explaining how a biological complex works. It is almost impossible for a single CART model to correctly explain an event without errors, even if it has all the useful and necessary variables. The principle of using several complementary trees then appears to be entirely judicious and appropriate. However, the assembly of complementary trees does not allow the specific effect of a variable to be evaluated when the effects of other variables have been taken into account. Breiman RFs, despite their statistical performance, are difficult to interpret from a technical point of view and pose a fundamental problem in describing a cascade of events. The complexity of a biological response often involves thousands of factors with multiple pathways of activation, and the result is quantitative information that, when a threshold effect comes into play, can be reduced to a binomial variable. However, it should be possible to introduce several dozen variables into each decision node. The advantage of a neuron is the ability to consider a very large number of input variables. A biological response could be modeled more effectively by putting a neuron into each node. In a previous article, we developed a new type of tree in response to this issue whose nodes consisted of a new type of neuron (Nguyen and Antonioli, submitted for publication; Nguyen and Antonioli, submitted for publication). In this model, each observation is classified at each node by a neuron as positive (false positive, FP, or true positive, TP) or negative (false negative, FN, or true negative, TN). Moreover, we created a new architecture of classification trees that allowed us to reinject a portion of the observations into the trunk. Indeed, we applied two independent inductions, one for negative observations (ŷ = 0) and another for positive observations that were systematically reinjected into the trunk (ŷ = 1), leading to a novel tree structure that forms a half Christmas tree structure.

We have demonstrated that the regression optimized (ROP) model exhibits very good diagnostic performance compared to other linear models and CART models (Nguyen and Antonioli, submitted for publication). Thus, it is interesting to compare the RF of ROP trees and the RF of Breiman CARTs.

We develop an RF strategy to analyze big data, including at least 30 explanatory variables, and propose a new variable selection method. In the first part, we focus on quantitative performance by comparing our results with those obtained by Breiman’s RF method, in particular with the Gini index. In the second part, we compare the genes selected and published in the GSE22513 dataset (Bauer *et al.*, 2010) and our gene selections.

## 2 Materials and methods

### 2.1 A new type of RF

To identify the most important predictive features, we developed a new class of information criteria to estimate the probability of a feature making a perfect prediction (PP) and how a PP can be achieved. We therefore go against the concepts of Breiman’s RFs by combining perfect trees (PTs) in massive quantities to approach the exhaustiveness of the possibilities and activation paths leading to an event. We define PTs as tree classifications without any classification errors, with a sensitivity and a specificity equal to 100%. For each forest, the number of variables included in each neuron of each tree is therefore fixed in advance. To build a tree, the variables are randomly selected from the variables in the database. Unlike in Breiman’s method, the observations are not bootstrapped. All observations are included in the analysis only once because bootstrapping would give different weights to the observations. Our strategy is to explore predictive features using only PTs. To reduce the risk of overfitting, we use PTs with a maximum of six nodes containing one neuron per node. In this way, we fix a predefined value to the depths of the trees. Therefore, no subsequent pruning needs to be performed. The number of trees in the forest depends on the number of features to be analyzed. We consider it necessary to have at least one hundred instances for each feature of the PTs being built.

The probability of obtaining a PT increases with the number of features included in each neuron and the range of simulation coefficients. The simulation range must be limited for the robustness of the model.

**Fig. 1.**
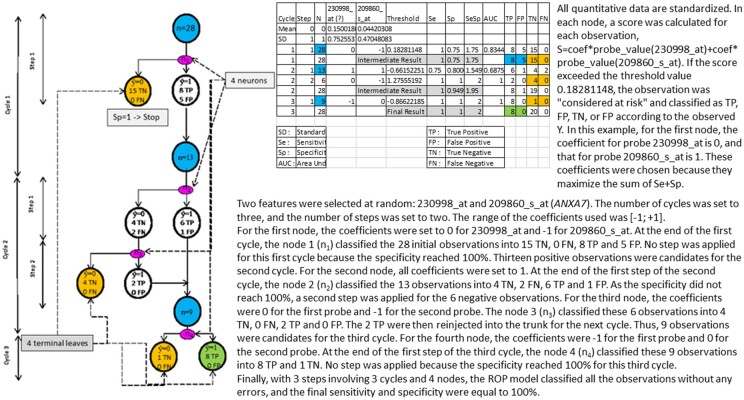
Example of a perfect tree including 2 probes [230998_at, 209860_s_at (ANXA7)] with the GSE22513 dataset.

**Fig. 2.**
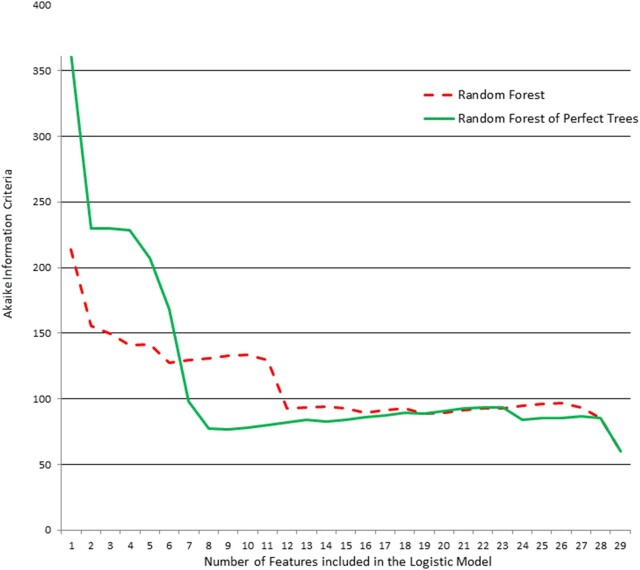
Breast cancer Wisconsin, dataset, features were intoduced into a LR model, acording to the Gini index or the NIC1 and the Akaike Information Criteria were compared.

A neuron including one variable would select variables very close to the studied state and would answer the question, ‘Which features are associated with the state?’ A neuron including more variables would select variables essential to the pathophysiological cascade leading to the state and would answer the question, ‘Which features are necessary to explain the physiological pathway?’ We developed strategies including 1, 2, or 3 selected features, and a fifteen-feature model was used to adjust and validate the previously selected features. The use of a simulation range (−1, 0, +1) facilitates the interpretation of the effects of the variables and reduces the calculation time. For each ROP tree, *n* features are randomized, and the prediction is performed on all observations with a range of (−1, 0, +1) for a tree with three cycles and two steps, which leads to the analysis of six neurons and 3^*n*^ combinations per neuron (Fig. 1). The number of features included is limited by the computation time per tree (Fig. 2).

### 2.2 Nguyen’s information criteria (NICs)

We have developed new statistical information criteria to describe and understand how a feature contributes to a perfect classification (Nguyen *et al.*, 2018) (Table 1).

**Table 1.** Nguyen’s information criteria (NICs)

Information typology	Information name	Definition	Estimation	Interpretation	Use
Information measuring the predictive performance of the variable for the event Y	NIC1	Probability for a variable to obtain a free-error prediction when it is associated with other variables	Ratio [number of occurrences where an error-free classification is obtained/number of times the variable is selected at random]	Maximum is the best. Estimate the probability to obtain an error-free classification if the classifier is in the neuron	Asses the predictive quality of the variable
	NIC2	Probability for a variable to have all coefficients of all neurons equal to zero when it is associated with other variables	Ratio [number of occurrences where all coefficients of all neurons are zero/number of times the variable is implied in an error-free classification]	Minimum is the best. Estimate the probability to obtain an error-free classification if all coefficients of the classifier are equal to zero	Assess the potential confounding effect of the variable
	NIC3	Probability for a variable to have all its coefficients positive in all neurons when it is associated with other variables	Ratio [number of occurrences where all coefficients of all neurons are positive/number of times the variable is implied in an error-free classification]	Estimate the increase of the risk associated with the classifier	Assess the increasing risk of the event Y associated with the variable
	NIC4	Probability for a variable to have all its coefficients negative in all neurons when it is associated with other variables	Ratio [number of occurrences where all coefficients of all neurons are negative/number of times the variable is implied in an error-free classification]	Estimate the decrease of the risk associated with the classifier	Assess the reduction risk of the event Y associated with the variable
	NIC5	Probability for a variable to have heterogeneous coefficients between trees (positive, negative, null) when it is associated with other variables	NIC3*NIC4	Minimum is the best. Estimate the probability that a variable may have a paradoxical effect	Assess the paradoxical effect of a variable
	NIC6	Probability for a variable to have heterogeneous coefficients within the same tree (positive, negative, null) when it is associated with other variables	Ratio [number of occurrences of the variable with coefficients not monotone/number of times the variable is implied in an error-free classification]	Estimate the probability of interaction of the classifier with other classifiers	Assess the potential interaction risk associated with the variable
Information measuring the proximity of the variable in the pathogenic path to the event Y	NIC7	Probability for a variable to achieve an error-free classification with only two neurons when it is associated with other variables	Ratio [number of occurrences of the variable where an error-free classification is obtained using only two neurons/number of times the variable is implied in an error-free classification]	Maximum is the best. Estimate the physiopathological distance between the classifier and the Y state	Assess the functional proximity of the variable and the event Y
	NIC8	Probability for a variable to achieve an error-free classification with only a single neuron when it is associated with other variables	Ratio [number of occurrences of the variable where an error-free classification is obtained using only a single neuron/number of times the variable is implied in an error-free classification]	Maximum is the best. Estimate a direct action of the classifier on the Y state	Assess the probability of a direct and linear relation between the variable and the event Y
Information measuring the complexity relationship between the variable and the event Y	NIC9	Probability for a variable to achieve an error-free classification with a finite number of solutions when it is associated with other variables	Ratio [number of occurrences where the classification tree has a number of enumerated solutions/number of times the variable is implied in an error-free classification]	Maximum is the best. Estimate the probability that the number of solutions is not infinite (no convergence of the model)	Assess the probability of a number of finites pathways between the variable and the event Y
	NIC10	Probability for a variable to achieve an error-free classification with a unique solution when it is associated with other variables	Ratio [number of occurrences where the classification tree has a unique solution/number of times the variable is implied in an error-free classification]	Maximum is the best. Estimate the probability that the solution is unique	Assess the probability of a unique pathway between the variable and the event Y

The first category represents the informative and predictive quality of a feature. The second category represents the information measuring the proximity of a feature in the pathogenic sequence to a state Y. The third category represents the information measuring the complexity of the relationship between a feature and state Y.

#### 2.2.1 Informative and predictive quality

The first criterion, NIC1, is defined as the probability that a feature will obtain an error-free prediction when it is associated with other features. NIC1 is estimated by the ratio of the number of occurrences for which an error-free classification is obtained/number of times the feature is selected at random.

The second criterion, NIC2, is defined as the probability that all the coefficients for all neurons of a feature will be equal to zero. NIC2 is estimated by the ratio of the number of occurrences for which all the coefficients for all neurons are equal to zero/number of times the feature is implied in an error-free classification. If all the coefficients for all neurons are equal to zero, the feature offers no independent predictive information.

Sometimes a feature can have all its positive coefficients in one tree (NIC3) and all its negative coefficients in another tree (NIC4). The effect of this type of feature changes depending on the presence of other factors, but its effect remains consistent for all observations in the same tree. We defined the NIC5 paradoxical criterion as the product of NIC3*NIC4. NIC5 measures the probability that a feature changes its effect as a function of other features. The features that are easiest to interpret are those with a zero probability.

This situation is different from that in which the coefficients of the same variable change sign in the same tree depending on the observations. The NIC6 criterion measures this variability.

Features with zero coefficients for all neurons are those that do not provide any discriminating information *a priori*. However, these features can also be intermediate features whose information is contained in the upstream and/or downstream features.

The most problematic features to interpret are those for which the signs of the coefficient vary in a tree (NIC6). These features increase or decrease the risk of state Y depending on the observation. By analyzing in detail the circumstances under which the coefficient of this type of feature changes its sign, we can identify the interactions of interest for this feature. A particular case is represented by the situation in which for all neurons, the feature has monotonic coefficients, except for one neuron whose coefficient has the opposite sign. This situation can then be more easily exploited. Indeed, if the coefficient of the feature changes its sign in only one neuron, then the observations involving this neuron contain information that the other observations do not contain. By extension, this situation is also applicable if the feature changes sign with respect to the mean effect expressed in the first neuron. This situation is the definition of what we call the ghost factor and has been described in another paper (Nguyen and Antonioli, submitted for publication) for which an application has already been published (Castillo *et al.*, 2016; Vildy et al., 2017).

We regard the best features as those that have the largest NIC1 and the smallest NIC2, NIC5 and NIC6.

#### 2.2.2 Proximity of a feature in the pathogenic sequence to state Y

We assume that the farther away a feature is from state Y, the more features are needed to obtain a PT. The first strategy is to count and identify the other features that are necessary to achieve an error-free prediction. The second and easiest strategy is to count the number of neurons required for this feature to be associated with a PT. We define NIC7 and NIC8 as the two-node model probability and one-node model probability, respectively.

We regard the best features as those that have the largest NIC7 and NIC8.

#### 2.2.3 Complexity of the relationship

In addition to the number of neurons and the number of features, the number of combinations that lead to the same PT is used as a measure of the potential for pathophysiological pathways. When the number of solutions is unique (NIC10), the interpretation is easy. On the other hand, several combinations (NIC9) or even an infinite number of combinations can lead to the same PT.

We regard the best features as those that have the largest NIC9 and NIC10.

We can now rank the variables by ranking these 10 criteria in order of importance. We can thus orient our classification by ranking variables according to the order of the values of the NICs. Several strategies can be discussed. We can give priority to performance by first ranking NIC1, followed by (1-NIC2), NIC8 and NIC10 (NIC1/(1-NIC2)/NIC10/NIC8) or first ranking (1-NIC2)/NIC1/NIC10/NIC8. This hierarchical strategy gives the first criterion major importance and minimizes the weight of the other criteria.

#### 2.2.4 NIC score

This hierarchical strategy would not allow us to consider all the NICs we have developed. Therefore, we propose a simple score that considers the three dimensions of the NICs, excluding NIC5 and NIC7, because NIC5 requires normalization into a new range from 0% to 100% and NIC7 is nested into NIC8. We penalize this score by NIC2 and NIC6, which assess the probability of null predictive information (NIC2) and the probability of a contrary effect (NIC6).

Finally, the NIC score is calculated as follows:

NICscore=NIC1−NIC2−NIC6+NIC8+NIC9+NIC10

#### 2.2.5 Datasets

##### 2.2.5.1 Breast Cancer Wisconsin (Diagnostic) dataset.

The Breast Cancer Wisconsin dataset, which includes 569 complete observations, was obtained from Kaggle (Blake and Merz, 1998). Ten real-valued features were computed for each cell nucleus. For each feature, the means, extrema and standard errors were collected, leading to 30 features.

##### 2.2.5.2 Markers of taxane sensitivity in breast cancer.

The public dataset GSE22513 involves expression profiling by Affymetrix^®^ arrays and includes fourteen patients with two replicates each and 54 675 features (Bauer *et al.*, 2010). The objective of this study was to identify molecular markers of the pathologic response to neoadjuvant paclitaxel/radiation treatment.

## 3 Algorithm

### 3.1 Feature selection

All variables were blinded during the full-time analysis and were renamed from a1 to a54765. An RF of perfect trees (RFPT) with one feature per neuron was developed for the learning step. Each feature was randomized at least 100 times. Features were ranked according to the NIC score and compared to the hierarchical ranking (1-NIC2)/NIC1/NIC10/NIC8. This comparison made it possible to evaluate the interest of a multidimensional score compared to a nested ranking.

### 3.2 Confirmation of selected features (Supplementary Data 3)

In the first step, we built three forests with trees that included 2, 3 or 15 randomized probes per neuron (adjusted approach). In the second step, we built a forest with trees that included 15 probes, 14 of which were unselected probes and one of which was a previously selected probe (stratified approach).

### 3.3 Comparison with the support vector machines—recursive feature elimination (SVM-RFE) method

The SVM-RFE method with a quadratic kernel was used to select informative genes in classification problems (Sanz et al., 2018). Threefold cross validation repeated 20 times was used to perform the RFE process. We used the Matthew Coefficient Correlation (MCC) and the coefficient of accuracy (CA) to compare the different prediction methods. Furthermore, several classifiers were built on the basis of each feature selection method: SVM, logistic regression (LR) and RF. We assessed the performance of each model by repeated cross validation.

## 4 Implementation

### 4.1 Diagnostic performance

The analysis used all 569 complete observations in the Breast Cancer Wisconsin dataset (Blake and Merz, 1998). For each tree, 15 of the 30 available features were selected at random. In total, 27 276 trees including 15 features selected at random were built, leading to 14 368 PTs (52.68%) (Supplementary Data 4).

Features were classified according to the Gini index or NIC1. At each step, the most important feature was introduced into a LR model, and the Akaike information criteria (AICs) were compared (Fig. 2). The LR models were compared using the ten most important features according to each information criterion (Supplementary Data 2). These results achieved better selection performance based on the AIC for the RFPT model than the RF model.

### 4.2 Real-world application

#### 4.2.1 Selection of probes using the NIC score

The results published in the literature with the GSE22513 dataset suggest that a pathological complete response (pCR) to paclitaxel was encountered in aggressive breast tumors and that an immune response and paclitaxel resistance were encountered in less aggressive tumors (Bauer *et al.*, 2010).

For the forest including one probe per node, 8 363 591 trees were built, of which 112 481 were PTs (1.34%). Each probe was, on average, selected at random 153 times. Among the 54 675 probes, 141 always led to PTs (NIC1 = 100%) that never had all null coefficients (NIC2 = 0%). Among these 141 probes, 65.24% (92/141) produced trees with a unique solution (NIC10 = 100%), and only 12 probes produced PTs with one node. Based on these results, the selection of the top 12 probes (*BBOX1 (205363_at), ZNF711 (207781_s_at), MAP2 (210015_s_at), CMC4 (210212_x_at), ADAMTS3 (214913_at), ACOT9 (221641_s_at), TOMM5 (225036_at, 228053_s_at), ERAP2 (227462_at), FAM3D (227676_at), OTULIN (228382_at), TLCD2 (241359_at))* was proposed. Table 2 shows the top 20 probes according to the hierarchical ranking (1-NIC2/NIC1/NIC10/NIC8).

**Table 2.** Top 20 probes according to hierarchical ranking (1-NIC2/NIC1/NIC10/NIC8)

Probe set ID	Gene symbol	Anonymized probes	Nb of trees	Nb of perfect trees (NIC1)	All coefficients = 0 (NIC2)	All coefficients positive (NIC3)	All coefficients negative (NIC4)	Other situations (NIC6)	Unique cycle (NIC7)	Unique neuron (NIC8)	Not solution at random (NIC9)	Unique solution (NIC10)	NICScore
**205363_at**	***BBOX1***	**a14811**	**160**	**160**	**0**	**160**	**0**	**0**	**160**	**160**	**160**	**160**	**4.00**
**207781_s_at**	***ZNF711***	**a17223**	**170**	**170**	**0**	**168**	**0**	**0**	**170**	**170**	**170**	**170**	**4.00**
**210015_s_at**	***MAP2***	**a19421**	**125**	**125**	**0**	**124**	**0**	**0**	**125**	**125**	**125**	**125**	**4.00**
**210212_x_at**	***CMC4***	**a19615**	**133**	**133**	**0**	**132**	**0**	**0**	**133**	**133**	**133**	**133**	**4.00**
**214913_at**	***ADAMTS3***	**a24209**	**144**	**144**	**0**	**143**	**0**	**0**	**144**	**144**	**144**	**144**	**4.00**
**221641_s_at**	***ACOT9***	**a30923**	**138**	**138**	**0**	**137**	**0**	**0**	**138**	**138**	**138**	**138**	**4.00**
**225036_at**	***TOMM5***	**a34294**	**159**	**159**	**0**	**158**	**0**	**0**	**159**	**159**	**159**	**159**	**4.00**
**227462_at**	***ERAP2***	**a36718**	**140**	**140**	**0**	**0**	**140**	**0**	**140**	**140**	**140**	**140**	**4.00**
**227676_at**	***FAM3D***	**a36931**	**138**	**138**	**0**	**138**	**0**	**0**	**138**	**138**	**138**	**138**	**4.00**
**228053_s_at**	***TOMM5***	**a37308**	**146**	**146**	**0**	**146**	**0**	**0**	**146**	**146**	**146**	**146**	**4.00**
**228382_at**	***OTULIN***	**a37637**	**149**	**149**	**0**	**147**	**0**	**0**	**149**	**149**	**149**	**149**	**4.00**
**241359_at**	***TLCD2***	**a50609**	**144**	**144**	**0**	**143**	**0**	**0**	**144**	**144**	**144**	**144**	**4.00**
1553875_s_at	*ZSCAN10*	a1172	155	155	0	0	0	155	0	0	155	155	2.00
1556008_a_at	*–*	a2731	174	174	0	0	0	174	0	0	174	174	2.00
200618_at	*LASP1*	a10067	177	177	0	0	0	177	0	0	177	177	2.00
200979_at	*PDHA1*	a10428	182	182	0	0	0	182	0	0	182	182	2.00
201220_x_at	*CTBP2*	a10669	164	164	0	0	0	164	0	0	164	164	2.00
202000_at	*NDUFA6*	a11449	159	159	0	0	0	159	0	0	159	159	2.00
202018_s_at	*LTF*	a11467	168	168	0	0	0	168	0	0	168	168	2.00
202382_s_at	*GNPDA1*	a11831	173	173	0	0	0	173	0	0	173	173	2.00

Selected features are highlighted in bold. Average = 153 times per probe—N Trees = 8,363,591; N Perfect Trees = 112,481 (1.34%).

The values correspond to the numerator of the ratios. The ratios are obtained by dividing each value by NIC1. Selection to the 12th feature is stopped because of the value of NIC8, which becomes null after the 12th feature.

Using the NIC score (Table 3), a gap was clearly identified between a group of 16 probes with a NIC score equal to 4.00 and all other probes, which had NIC scores of 2.00 or less. The top 12 probes were identical to the top 12 probes previously identified using the hierarchical ranking method of selection. Fifteen of these probes (*PDHA1 (200980_s_at), VBP1 (201472_at), BBOX1 (205363_at), ZNF711 (207781_s_at), MAP2 (210015_s_at), CMC4 (210212_x_at), ADAMTS3 (214913_at), IFT81 (219372_at), ACOT9 (221641_s_at), CLDN12 (223249_at), TOMM5 (225036_at, 228053_s_at)), ERAP2 (227462_at)*, *FAM3D (227676_at), OTULIN (228382_at)*) were concordant with those reported in the papers by Ali *et al.* (2014) and Bauer *et al.* (2010) and/or the hypothesis that high tumor aggressiveness is associated with a high level of pCR, and conversely, low tumor aggressiveness is associated with a low level of pCR. Limited biological information was available for the last probe, *TLCD2 (241359_at).*

**Table 3.** Top 20 probes ranked according to NICScore

Probe set ID	Gene symbol	Nb of trees	Nb of perfect trees (NIC1)	All coefficients = 0 (NIC2)	All coefficients positive (NIC3)	All coefficients negative (NIC4)	Other situations (NIC6)	Unique cycle (NIC7)	Unique neuron (NIC8)	Not solution at random (NIC9)	Unique solution (NIC10)	NICScore
**205363_at**	***BBOX1***	**160**	**160**	**160**	**0**	**160**	**0**	**0**	**160**	**160**	**160**	**4.00**
**207781_s_at**	***ZNF711***	**170**	**170**	**170**	**0**	**168**	**0**	**0**	**170**	**170**	**170**	**4.00**
**210015_s_at**	***MAP2***	**125**	**125**	**125**	**0**	**124**	**0**	**0**	**125**	**125**	**125**	**4.00**
**210212_x_at**	***CMC4***	**133**	**133**	**133**	**0**	**132**	**0**	**0**	**133**	**133**	**133**	**4.00**
**214913_at**	***ADAMTS3***	**144**	**144**	**144**	**0**	**143**	**0**	**0**	**144**	**144**	**144**	**4.00**
**221641_s_at**	***ACOT9***	**138**	**138**	**138**	**0**	**137**	**0**	**0**	**138**	**138**	**138**	**4.00**
**225036_at**	***TOMM5***	**159**	**159**	**159**	**0**	**158**	**0**	**0**	**159**	**159**	**159**	**4.00**
**227462_at**	***ERAP2***	**140**	**140**	**140**	**0**	**0**	**140**	**0**	**140**	**140**	**140**	**4.00**
**227676_at**	***FAM3D***	**138**	**138**	**138**	**0**	**138**	**0**	**0**	**138**	**138**	**138**	**4.00**
**228053_s_at**	***TOMM5***	**146**	**146**	**146**	**0**	**146**	**0**	**0**	**146**	**146**	**146**	**4.00**
**228382_at**	***OTULIN***	**149**	**149**	**149**	**0**	**147**	**0**	**0**	**149**	**149**	**149**	**4.00**
**241359_at**	***TLCD2***	**144**	**144**	**144**	**0**	**143**	**0**	**0**	**144**	**144**	**144**	**4.00**
**223249_at**	***CLDN12***	**154**	**154**	**154**	**0**	**0**	**153**	**0**	**154**	**154**	**154**	**3.99**
**201472_at**	***VBP1***	**153**	**153**	**152**	**0**	**152**	**0**	**0**	**152**	**152**	**152**	**3.99**
**200980_s_at**	***PDHA1***	**152**	**152**	**151**	**0**	**150**	**0**	**0**	**151**	**151**	**151**	**3.99**
**219372_at**	***IFT81***	**146**	**146**	**146**	**0**	**0**	**145**	**0**	**146**	**146**	**146**	**3.99**
1553875_s_at	*ZSCAN10*	155	155	155	0	0	0	155	0	0	155	2.00
1556008_a_at	*–*	174	174	174	0	0	0	174	0	0	174	2.00
200618_at	*LASP1*	177	177	177	0	0	0	177	0	0	177	2.00
200979_at	*PDHA1*	182	182	182	0	0	0	182	0	0	182	2.00

Selected features are highlighted in bold. The first 16 features were selected because the 17th feature had a very important NICScore difference.

Average =153 times per probe—N Trees = 8,363,591; N Perfect Trees = 112,481 (1.34%).

These results were confirmed using adjusted and stratified analyses (Supplementary Data 3).

#### 4.2.2 Comparison with the SVM-RFE method

SVM-RFE with a quadratic kernel was used to select informative genes in classification problems (Sanz *et al.*, 2018). Threefold cross validation repeated 20 times was used to perform the RFE process. We used the MCC and the CA to compare the different prediction methods. Furthermore, several classifiers were built on the basis of each feature selection method: SVM, LR, and RF. We assessed the performance of each model by repeated cross validation. The 15 optimal probes selected by this method were included in the top 16 probes of the RFPT-NIC score (Table 4, Supplementary Data 4). The results showed that the top 16 probes selected by the NIC score were a slight better than those proposed by the RFE-SVM procedure, MCC_SVM-RFE_ =77.2% +/− 27.0% vs MCC_RFPT-NIC score_ = 77.4%+/−18.8%; CA_RFE-SVM_ =88.64%+/−12.1% vs CA_RFPT-NIC score_ = 89.5%+/−9.3%).

**Table 4.** Selection of probes using the SVM-RFE procedure compared to the RFPT-NIC score

Number of probes	SVM-RFE		RFPT-NICs score	
	Probe set ID	Gene symbol	Probe set ID	Gene symbol
1	200980_s_at	*PDHA1*	205363_at	*BBOX1*
2	201472_at	*VBP1*	207781_s_at	*ZNF711*
3	205363_at	*BBOX1*	210015_s_at	*MAP2*
4	207781_s_at	*ZNF711*	210212_x_at	*CMC4*
5	210015_s_at	*MAP2*	214913_at	*ADAMTS3*
6	210212_x_at	*CMC4*	221641_s_at	*ACOT9*
7	214913_at	*ADAMTS3*	225036_at	*TOMM5*
8	219372_at	*IFT81*	227462_at	*ERAP2*
9	221641_s_at	*ACOT9*	227676_at	*FAM3D*
10	223249_at	*CLDN12*	228053_s_at	*TOMM5*
11	225036_at	*TOMM5*	228382_at	*OTULIN*
12	227462_at	*ERAP2*	241359_at	*TLCD2*
13	227676_at	*FAM3D*	223249_at	*CLDN12*
14	228053_s_at	*TOMM5*	201472_at	*VBP1*
15	228382_at	*OTULIN*	200980_s_at	*PDHA1*
16	241359_at	*TLCD2*	219372_at	*IFT81*

The two methods selected the same top 16 probes. Only the ranks were different.

The comparison of the three top probe lists also used LR, RF and SVM. The results showed that the metrics across the three models were consistently high, with a slight advantage for the RFPT_NICs score method (Supplementary Data 5).

## 5 Discussion

We developed a new paradigm for RFs that differs from Breiman’s approach, which is a forest that includes only perfectly classified trees (Nguyen and Antonioli, submitted for publication).

Using a neuron in each node, we simultaneously adjusted the effects of each feature relative to other features into a decision tree.

These innovations were accompanied by the development of new statistical information criteria (NICs) and a score (NIC score) that takes into account the three dimensions (performance, proximity and simplicity) explored by these NICs.

We used different datasets to analyze the performance and reproducibility of the feature selection and illustrated the use of this new approach with a complex omics dataset.

A biochemical reaction involving molecules is written as a perfect model that has no room for an error term because there is no variability in perfectly identified molecules and all the information is known. Therefore, the idea was to start from known results and then describe and assemble the combinatorial possibilities to obtain results without making any errors. The problem is the exponential Exp(n) number of combinations to be analyzed, where n is the number of variables, and randomness is used to minimize bias.

NICs are PT ratios, and thus, the amount of information that can be analyzed increases with the number of features included in the neurons. In the example of GSE22513 breast cancer data, this probability was 1.34% with one feature and exceeded 99% with 15 variables per neuron. The possibility of obtaining a large quantity of PTs was found in all the databases we analyzed. These results have at least two consequences. The first is that this perfect tree approach is possible as long as we have enough features. The second is that it is also possible to conclude that the data are insufficient or inappropriate for drawing conclusions when PTs cannot be produced. However, although the AIC was suitable for the statistical model, our objective was to explain a situation without making errors rather than applying parsimony of statistical models.

To increase the robustness and precision of selection, we used only 3 coefficients, negative, zero or positive, which allowed for direct interpretation of the effects of the variables. The restriction to only 3 coefficients also reduced the computation time. Thus, this method allowed us to determine whether one feature had an agonistic or antagonistic effect relative to the action of the other features. In another paper, we explained that a change in the sign of the coefficient for the same feature could be interpreted as a differential effect between clusters of observations, and these situations were used to identify gap information, which is called the ghost factor (Nguyen and Antonioli submitted for publication; Vildy *et al.*, 2017). The consequence of such results is the ability to fill in missing information and address a kind of interaction mapping between features, and these interactions can be considered physiological pathways. In doing so, we expected to create a new method that allows us to understand different possible pathophysiological pathways. These associations may correspond to physiopathological pathways that will have to be verified experimentally. The methodology of this strategy is under development, and the results will be presented in another work.

The classic approach to deep learning begins with a contribution and continues, layer by layer, without the ability to return to previous layers. The ROP model replaces deep learning with an RF. The complex set ‘neuron-ROP-RPTF’ can be considered a competing process in the deep layers. In contrast to deep learning, the procedure for selecting features and their effects is completely readable and understandable, which makes it possible to understand the effects of each feature and compare them with existing data. The main limitation of the model is that it considers all data to be exact and does not support missing data, as with most multivariate models. No existing imputation method is acceptable for the ROP model, as it is a combinatorial analysis in which the notion of a central value is not relevant. Any inaccuracies in the data will be reflected in the results. Our model seeks to explain a state to understand the mechanisms involved and their respective influences. One of the important questions we ask ourselves concerns our feature selection strategy. A hierarchical procedure was used to rank the features, which resulted in the first criterion having too much of an influence on the others, so the composite NIC score was developed. The results showed that the performance criterion (1-NIC2) alone does not allow the information associated with the importance of a variable to be taken into account.

An important issue concerns the features identified at least once as always having null coefficients in all PT neurons. From a statistical point of view, these features have no value. However, we do not employ a prediction model but rather an explanatory model. These features can simply be intermediate features that may be essential, or even limiting, features in the cascade of events. It is therefore very dangerous to eliminate these features from subsequent analyses.

This phenomenon is why analyses should start with only one variable per neuron to measure the potential of each variable independently of others. Thus, we were able to verify that in the presence of one of the 16 important and selected variables in the GSE22513 dataset, the coefficients of the 15 other variables could all be equal to zero in a perfect tree.

However, if neurons containing one feature allow us to first select candidates, as in bivariate analysis, then neurons with several features allow us to confirm predictive features after adjusting for other features. Another advantage of the use of neurons that include multiple features is the identification of associations of features that, when taken together, would provide the best possible prediction. Multivariate neurons provide the possibility of describing, identifying and understanding the circumstances required to obtain a perfect tree, which is an important issue in the interpretation of the set of features used to modulate a target feature. We believe this is a very good strategy for understanding the mechanism of how a feature works. The ROP tree allows us to directly see the effect of each feature and identify the associations that lead to error-free classifications. This approach makes it possible to distinguish between what is common from one set of features specific to another set, particularly what modulator features are associated with features that increase or decrease the sensitivity to paclitaxel. This work is ongoing.

Validation of the results obtained by the RFPT-NIC_score was successfully conducted by comparing the selection and prediction of features with other methods, such as the RFE-SVM procedure. The results showed that the performance of our approach presented a slight advantage to that of the RFE-SVM procedure. However, more datasets have to be used to better quantify the potential gain.

Beyond the quantitative aspect of performance, we have opened up a field of research into criteria for evaluating the pathophysiological proximity of features to a biological event of interest and the complexity of their relationships. With these two pieces of information, we were able to propose a mapping of the cascade of features leading to the event of interest. The mechanism explaining the effectiveness of paclitaxel is not fully understood (Lohard *et al.*, 2020; Weaver, 2014). Our analysis focused on the genes presented by the authors, and all the selected features were concordant with the hypothesis that high tumor aggressiveness is associated with a high level of pCR and, conversely, that low tumor aggressiveness is associated with a low level of pCR. Our analysis demonstrated that the probes and genes we selected using the initial 2010 dataset were fully consistent with the current hypotheses of paclitaxel resistance, although they had not been identified by the authors in 2010. We also identified a promising new probe for which limited biological information (*TLCD2*) was available. Based on our results, this probe (241359_at) should be biologically explored to confirm its role in taxane sensitivity.

The modifications concern the neuron itself and include new functions regarding conditioning activation and changes in the propagation mode. These new architectures will make it possible to discover new combinations of features and thus enrich the model information. Another step is to improve the development of the NIC score. However, the major change will involve the use of the convolutional neural network architecture of deep learning by using patterns to summarize small chains of pathophysiological cascades involving at most two levels (i.e., a variable and its covariates involved in a perfect tree). In conclusion, we have demonstrated that the numerical and combinatorial approach used by the RFPT model is an excellent solution for understanding the role of factors involved in explaining an event, which opens up multiple perspectives.

## Editor

We submitted this manuscript to an independent professional editor (American Journal Experts) to ensure that the text was edited prior to submission.

## Supplementary Material

btab074_Supplementary_Data
