# Supplementary material for: Random forest of perfect trees: concept, performance, applications and perspectives
Source: Bioinformatics. 2021 Feb 1;37(15):2165–74. doi: 10.1093/bioinformatics/btab074 (PMC8352507; doi:10.1093/bioinformatics/btab074)
Supplement: btab074_Supplementary_Data [file btab074_supplementary_data.zip › supplementary data 1 revision2.docx]

Supplementary data 1 : Confirmatory analyses

1. Adjusted analyses

In this analysis, all probes were randomly added to a neuron which could include one or more selected probes, in order to compare the results with RFPT containing only one variable per neuron.

1.1 For the forest, for which 2 randomized probes were included per node, each probe was, on average, selected at random 710 times, leading to the construction of 19 401 295 trees, of which 2 237 948 were perfect trees (11.53%). Among the 54 675 probes, only 5 always led to perfect trees (NIC1=100%) that never had all null coefficients (NIC2=0%).

According to the hierarchical ranking method, the 12 previously selected probes were ranked within the top 20. Probe 227462_at (*ERAP2*) was not included in the top 20. Four probes that were not initially identified by the hierarchical method but were identified by the NICscore were now included in the top 16.

According to the NICscore method, all of the probes that were initially ranked in the top 16 were again in the top 16 at this step.

Confirmation using adjusted analysis with 2 features using by the hierarchical ranking.

Each variable was selected at random an average of 710 times. A forest of 19 401 295 trees was constructed in which 2 237 948 were perfect trees (11.53%).

The 12 features initially selected by the hierarchical method are highlighted in yellow, while the features initially selected by the NICScore were highlighted in green. These adjusted models showed that the four variables that were initially identified by the NICScore method but not by the hierarchical method, were now in the top 12.

| Probe Set ID | Gene Symbol | Nb of trees | NIC1 | NIC2 | NIC3 | NIC4 | NIC6 | NIC7 | NIC8 | NIC9 | NIC10 | NICScore |
| --- | --- | --- | --- | --- | --- | --- | --- | --- | --- | --- | --- | --- |
| 227676_at | *FAM3D* | 763 | 763 | 0 | 763 | 0 | 0 | 763 | 763 | 763 | 731 | 3.958 |
| 241359_at | *TLCD2* | 791 | 791 | 0 | 791 | 0 | 0 | 791 | 791 | 791 | 756 | 3.956 |
| 210212_x_at | *CMC4* | 750 | 750 | 0 | 750 | 0 | 0 | 750 | 750 | 750 | 692 | 3.923 |
| 223249_at | *CLDN12* | 759 | 759 | 0 | 0 | 758 | 0 | 759 | 759 | 759 | 685 | 3.903 |
| 210015_s_at | *MAP2* | 722 | 722 | 0 | 722 | 0 | 0 | 722 | 722 | 722 | 647 | 3.896 |
| 200980_s_at | *PDHA1* | 799 | 798 | 0 | 798 | 0 | 0 | 798 | 798 | 798 | 761 | 3.952 |
| 212531_at | *LCN2* | 771 | 770 | 0 | 2 | 0 | 768 | 2 | 2 | 770 | 581 | 1.758 |
| 219372_at | *IFT81* | 758 | 757 | 0 | 0 | 756 | 0 | 757 | 757 | 757 | 725 | 3.956 |
| 201472_at | *VBP1* | 758 | 757 | 0 | 757 | 0 | 0 | 757 | 757 | 757 | 683 | 3.901 |
| 205363_at | *BBOX1* | 756 | 755 | 0 | 755 | 0 | 0 | 755 | 755 | 755 | 729 | 3.964 |
| 223634_at | *RASD2* | 728 | 727 | 0 | 4 | 0 | 723 | 2 | 2 | 727 | 182 | 1.257 |
| 225036_at | *TOMM5* | 723 | 722 | 0 | 721 | 0 | 0 | 722 | 722 | 722 | 657 | 3.909 |
| 228053_s_at | *TOMM5* | 722 | 721 | 0 | 721 | 0 | 0 | 721 | 721 | 721 | 673 | 3.932 |
| 210139_s_at | *PMP22* | 675 | 674 | 0 | 0 | 0 | 674 | 649 | 0 | 674 | 345 | 1.510 |
| 221641_s_at | *ACOT9* | 763 | 761 | 0 | 761 | 0 | 0 | 761 | 761 | 761 | 736 | 3.965 |
| 207781_s_at | *ZNF711* | 761 | 759 | 0 | 758 | 0 | 0 | 759 | 759 | 759 | 736 | 3.967 |
| 228382_at | *OTULIN* | 749 | 747 | 0 | 747 | 0 | 0 | 747 | 747 | 747 | 712 | 3.950 |
| 205182_s_at | *ZNF324* | 749 | 747 | 0 | 38 | 0 | 709 | 19 | 19 | 747 | 329 | 1.514 |
| 202560_s_at | *C1orf77* | 737 | 735 | 0 | 1 | 0 | 734 | 1 | 1 | 735 | 631 | 1.859 |
| 214913_at | *ADAMTS3* | 808 | 805 | 0 | 805 | 0 | 0 | 805 | 805 | 805 | 784 | 3.970 |

1.2 RFPT with 3 or 15 randomized probes per node.

For the forest, when we included 3 or 15 randomized probes per node, we observed exactly the same results that we did with the adjusted model with 2 features per node. The NICScore allowed us to select the top 16 probes with high reproducibility from among the different adjusted models. The hierarchical method presented greater variability and did not include the initially selected top 16 probes.

1.2.1 Confirmation using adjusted analysis with 3 features using by the hierarchical ranking.

Each variable was selected at random an average of 942 times. A forest of 17 161 331 trees was constructed in which 7 156 233 were perfect trees (41.7%). This analysis confirmed the results of the 2-variable per neuron model: the 4 variables identified by the NICScore now appear in the list of the 12 most important variables.

| Probe Set ID | Gene Symbol | Nb of trees | NIC1 | NIC2 | NIC3 | NIC4 | NIC6 | NIC7 | NIC8 | NIC9 | NIC10 | NICScore |
| --- | --- | --- | --- | --- | --- | --- | --- | --- | --- | --- | --- | --- |
| 210015_s_at | *MAP2* | 962 | 961 | 0 | 961 | 0 | 0 | 961 | 961 | 961 | 745 | 3.774 |
| 205363_at | *BBOX1* | 990 | 988 | 0 | 988 | 0 | 0 | 988 | 988 | 988 | 877 | 3.886 |
| 200980_s_at | *PDHA1* | 975 | 973 | 0 | 973 | 0 | 0 | 973 | 973 | 973 | 886 | 3.909 |
| 225036_at | *TOMM5* | 946 | 944 | 0 | 944 | 0 | 0 | 944 | 944 | 944 | 771 | 3.815 |
| 201472_at | *VBP1* | 938 | 936 | 0 | 936 | 0 | 0 | 936 | 936 | 936 | 771 | 3.822 |
| 228053_s_at | *TOMM5* | 995 | 992 | 0 | 992 | 0 | 0 | 992 | 992 | 992 | 862 | 3.866 |
| 210212_x_at | *CMC4* | 963 | 960 | 0 | 960 | 0 | 0 | 960 | 960 | 960 | 804 | 3.834 |
| 219372_at | *IFT81* | 946 | 943 | 0 | 0 | 943 | 0 | 943 | 943 | 943 | 837 | 3.884 |
| 223249_at | *CLDN12* | 976 | 972 | 0 | 0 | 971 | 0 | 972 | 972 | 972 | 739 | 3.756 |
| 227676_at | *FAM3D* | 976 | 971 | 0 | 971 | 0 | 0 | 971 | 971 | 971 | 886 | 3.907 |
| 241359_at | *TLCD2* | 978 | 972 | 0 | 972 | 0 | 0 | 972 | 972 | 972 | 876 | 3.895 |
| 207781_s_at | *ZNF711* | 959 | 953 | 0 | 953 | 0 | 0 | 953 | 953 | 953 | 879 | 3.916 |
| 214913_at | *ADAMTS3* | 978 | 971 | 0 | 971 | 0 | 0 | 971 | 971 | 971 | 903 | 3.923 |
| 228382_at | *OTULIN* | 949 | 942 | 0 | 942 | 0 | 0 | 942 | 942 | 942 | 870 | 3.916 |
| 212100_s_at | *POLDIP3* | 955 | 944 | 0 | 13 | 0 | 931 | 5 | 5 | 944 | 131 | 1.146 |
| 205830_at | *CLGN* | 972 | 956 | 0 | 0 | 32 | 924 | 13 | 13 | 956 | 145 | 1.182 |
| 204657_s_at | *SHB* | 948 | 886 | 0 | 280 | 0 | 606 | 30 | 29 | 886 | 68 | 1.360 |
| 229470_at | *---* | 941 | 877 | 0 | 74 | 0 | 803 | 37 | 37 | 877 | 170 | 1.252 |
| 232739_at | *SPIB* | 929 | 861 | 0 | 75 | 0 | 786 | 33 | 32 | 861 | 115 | 1.185 |
| 202134_s_at | *WWTR1* | 984 | 911 | 0 | 402 | 0 | 509 | 69 | 69 | 911 | 267 | 1.736 |

1.2.2 Confirmation using adjusted analysis with 15 features using by the hierarchical ranking.

Each variable was selected at random an average of 293 times. A forest of 1 068 700 trees was constructed in which 1 064 030 were perfect trees (99.6%). This analysis confirmed the results of the 2-variable per neuron model: the 4 variables identified by the NICScore now appear in the list of the 16 most important variables.

| Probe Set ID | Gene Symbol | Nb of trees | NIC1 | NIC2 | NIC3 | NIC4 | NIC6 | NIC7 | NIC8 | NIC9 | NIC10 | NICScore |
| --- | --- | --- | --- | --- | --- | --- | --- | --- | --- | --- | --- | --- |
| 207781_s_at | *ZNF711* | 312 | 311 | 0 | 311 | 0 | 0 | 311 | 311 | 311 | 1 | 3.000 |
| 219372_at | *IFT81* | 309 | 308 | 0 | 0 | 308 | 0 | 308 | 308 | 308 | 2 | 3.003 |
| 210015_s_at | *MAP2* | 305 | 304 | 0 | 304 | 0 | 0 | 304 | 304 | 304 | 0 | 2.997 |
| 210212_x_at | *CMC4* | 276 | 275 | 0 | 275 | 0 | 0 | 275 | 275 | 275 | 2 | 3.004 |
| 227462_at | *ERAP2* | 328 | 326 | 0 | 0 | 326 | 0 | 325 | 325 | 326 | 0 | 2.991 |
| 228053_s_at | *TOMM5* | 314 | 312 | 0 | 310 | 2 | 0 | 309 | 309 | 311 | 1 | 2.984 |
| 227676_at | *FAM3D* | 327 | 325 | 1 | 324 | 0 | 0 | 325 | 325 | 325 | 0 | 2.991 |
| 228382_at | *OTULIN* | 319 | 315 | 1 | 314 | 0 | 0 | 315 | 315 | 315 | 6 | 3.003 |
| 205363_at | *BBOX1* | 299 | 298 | 1 | 296 | 1 | 0 | 297 | 297 | 298 | 3 | 3.000 |
| 200980_s_at | *PDHA1* | 278 | 277 | 1 | 276 | 0 | 0 | 277 | 277 | 277 | 1 | 2.996 |
| 223249_at | *CLDN12* | 273 | 271 | 1 | 0 | 270 | 0 | 271 | 271 | 271 | 1 | 2.993 |
| 225036_at | *TOMM5* | 269 | 267 | 1 | 266 | 0 | 0 | 267 | 267 | 267 | 2 | 2.996 |
| 241359_at | *TLCD2* | 287 | 287 | 2 | 285 | 0 | 0 | 287 | 287 | 287 | 3 | 3.003 |
| 201472_at | *VBP1* | 287 | 285 | 2 | 283 | 0 | 0 | 285 | 285 | 285 | 0 | 2.986 |
| 214913_at | *ADAMTS3* | 311 | 310 | 3 | 307 | 0 | 0 | 310 | 310 | 310 | 8 | 3.013 |
| 221641_s_at | *ACOT9* | 310 | 309 | 3 | 305 | 0 | 1 | 308 | 308 | 309 | 2 | 2.987 |
| 212416_at | *SCAMP1* | 281 | 281 | 4 | 0 | 275 | 2 | 278 | 278 | 281 | 0 | 2.968 |
| 204567_s_at | *ABCG1* | 309 | 308 | 5 | 0 | 299 | 4 | 304 | 304 | 308 | 1 | 2.958 |
| 203502_at | *BPGM* | 289 | 289 | 5 | 0 | 283 | 1 | 287 | 287 | 289 | 0 | 2.972 |
| 235573_at | --- | 275 | 274 | 5 | 0 | 265 | 4 | 269 | 269 | 273 | 0 | 2.942 |

1. Confirmation using stratified analyses

In this analysis, neurons were stratified on each selected probe. Fourteen probes were randomly added, along with one of the selected probes. All neurons included exactly one selected probe. A forest was built based on the 12 top probes selected with the hierarchical method and another forest based on the 16 top probes selected by the NICScore. Therefore, selected probes were not adjusted on another selected probe and consequently were not in competition with another selected probe. The expected result was that we had to get the initially selected probes into the group of top probes selected by each strategy.

The top 12 probes selected by hierarchical selection confirmed the initially selected 12 probes. However, in this confirmatory analysis, the remaining 4 items identified by the NICScore method were ranked just after the 12 stratified items. Moreover, among the non-stratified items, there was a significant discrepancy in the NIC2 criterion, and even in the NICScore, between these 4 items and the other non-stratified items. This showed that the prioritized method did not identify these 4 important items.

The top 16 probes selected by the NICScore were again ranked among the top 16 probes. Moreover, there was a gap in NICScore between the top 16 probes (mean=4.0 +/- 0.005, min=3.99, max=4.01) and the other probes (mean=3.01+/-0.008, min=2.79, max=3.05) (Table 6b).

2.1 Confirmation using stratified analysis for features initially selected by hierarchical ranking

Each variable was selected at random an average of 207 times. A forest of 756 188 trees was constructed in which 755 192 were perfect trees (99.9%).

The 4 items identified by the NICScore method were ranked just after the 12 stratified items. Among the non-stratified items, there was a significant discrepancy in the NIC2 criterion and the NICScore between these 4 items and the other non-stratified items.

| Probe Set ID | Gene Symbol | Nb of trees | NIC1 | NIC2 | NIC3 | NIC4 | NIC6 | NIC7 | NIC8 | NIC9 | NIC10 | NICScore |
| --- | --- | --- | --- | --- | --- | --- | --- | --- | --- | --- | --- | --- |
| 241359_at | *TLCD2* | 56678 | 56667 | 41 | 56603 | 7 | 4 | 56655 | 56655 | 56666 | 385 | 4.006 |
| 210212_x_at | *CMC4* | 28174 | 28168 | 34 | 28118 | 5 | 4 | 28157 | 28157 | 28167 | 428 | 4.013 |
| 227676_at | *FAM3D* | 71723 | 71700 | 136 | 71531 | 8 | 12 | 71649 | 71649 | 71700 | 955 | 4.010 |
| 227462_at | *ERAP2* | 53142 | 53022 | 118 | 2 | 52702 | 26 | 52978 | 52978 | 53019 | 164 | 3.997 |
| 210015_s_at | *MAP2* | 76979 | 76967 | 172 | 76766 | 6 | 10 | 76913 | 76913 | 76963 | 358 | 4.001 |
| 207781_s_at | *ZNF711* | 69448 | 69322 | 178 | 68835 | 130 | 25 | 69151 | 69150 | 69305 | 446 | 3.999 |
| 221641_s_at | *ACOT9* | 54685 | 54564 | 156 | 54134 | 114 | 25 | 54414 | 54411 | 54543 | 982 | 4.009 |
| 228382_at | *OTULIN* | 100904 | 1E+05 | 297 | 1E+05 | 83 | 34 | 1E+05 | 1E+05 | 1E+05 | 1472 | 4.009 |
| 214913_at | *ADAMTS3* | 69107 | 68954 | 205 | 68465 | 107 | 24 | 68795 | 68795 | 68942 | 855 | 4.004 |
| 225036_at | *TOMM5* | 53173 | 53053 | 161 | 52626 | 115 | 22 | 52895 | 52892 | 53039 | 610 | 4.002 |
| 228053_s_at | *TOMM5* | 51753 | 51747 | 163 | 51545 | 13 | 12 | 51682 | 51682 | 51745 | 749 | 4.010 |
| 205363_at | *BBOX1* | 70430 | 70217 | 301 | 69486 | 188 | 55 | 69932 | 69927 | 70194 | 363 | 3.993 |
| 223249_at | *CLDN12* | 170 | 170 | 55 | 0 | 114 | 1 | 169 | 169 | 170 | 0 | 3.665 |
| 219372_at | *IFT81* | 199 | 198 | 75 | 0 | 123 | 0 | 198 | 198 | 198 | 0 | 3.616 |
| 200980_s_at | *PDHA1* | 195 | 195 | 83 | 105 | 7 | 0 | 195 | 195 | 195 | 1 | 3.579 |
| 201472_at | *VBP1* | 158 | 158 | 71 | 86 | 1 | 0 | 158 | 158 | 158 | 1 | 3.557 |
| 225336_at | *SFRS2IP* | 172 | 172 | 165 | 0 | 7 | 0 | 172 | 172 | 172 | 0 | 3.041 |
| 215152_at | *MYB* | 174 | 174 | 167 | 0 | 6 | 1 | 173 | 173 | 174 | 1 | 3.034 |
| 239033_at | *---* | 205 | 204 | 196 | 0 | 8 | 0 | 202 | 202 | 204 | 0 | 3.025 |
| 228354_at | *C10orf83* | 194 | 194 | 187 | 0 | 6 | 1 | 193 | 193 | 194 | 0 | 3.026 |

2.2 Confirmation using stratified analysis for features initially selected by the NICScore

Each variable was selected at random an average of 350 times. A random forest trees was constructed in which 1 275 481 were perfect trees 1 274 024 (99.9%).

The 16 features initially selected by NICScore are highlighted in green. These 16 features were ranked among the top 16 variables in this stratified analysis. Note that there is a significant difference in the NICScore between the first 16 features selected and the other features.

| Probe Set ID | Gene Symbol | Nb of trees | NIC1 | NIC2 | NIC3 | NIC4 | NIC6 | NIC7 | NIC8 | NIC9 | NIC10 | NICScore |
| --- | --- | --- | --- | --- | --- | --- | --- | --- | --- | --- | --- | --- |
| 228382_at | *OTULIN* | 77120 | 77092 | 236 | 76772 | 28 | 22 | 76986 | 76986 | 77078 | 1118 | 4.009 |
| 214913_at | *ADAMTS3* | 1E+05 | 1E+05 | 303 | 1E+05 | 152 | 40 | 1E+05 | 1E+05 | 1E+05 | 1616 | 4.008 |
| 241359_at | *TLCD2* | 86304 | 86302 | 166 | 86122 | 0 | 14 | 86238 | 86238 | 86302 | 918 | 4.008 |
| 207781_s_at | *ZNF711* | 65030 | 65026 | 48 | 64961 | 6 | 11 | 64993 | 64991 | 65026 | 466 | 4.006 |
| 221641_s_at | *ACOT9* | 1E+05 | 1E+05 | 158 | 1E+05 | 95 | 21 | 1E+05 | 1E+05 | 1E+05 | 1081 | 4.005 |
| 210212_x_at | *CMC4* | 1E+05 | 1E+05 | 194 | 1E+05 | 102 | 30 | 1E+05 | 1E+05 | 1E+05 | 1052 | 4.005 |
| 200980_s_at | *PDHA1* | 53055 | 53021 | 110 | 52818 | 31 | 20 | 52956 | 52955 | 53016 | 377 | 4.003 |
| 205363_at | *BBOX1* | 56545 | 56523 | 72 | 56413 | 18 | 4 | 56486 | 56486 | 56523 | 268 | 4.002 |
| 219372_at | *IFT81* | 1E+05 | 1E+05 | 236 | 1 | 1E+05 | 66 | 1E+05 | 1E+05 | 1E+05 | 661 | 4.001 |
| 227676_at | *FAM3D* | 63835 | 63741 | 155 | 63358 | 86 | 32 | 63592 | 63592 | 63717 | 455 | 4.000 |
| 201472_at | *VBP1* | 99299 | 99216 | 163 | 98937 | 53 | 18 | 99108 | 99107 | 99211 | 301 | 3.999 |
| 210015_s_at | *MAP2* | 1E+05 | 1E+05 | 325 | 1E+05 | 73 | 26 | 1E+05 | 1E+05 | 1E+05 | 512 | 3.999 |
| 227462_at | *ERAP2* | 56824 | 56706 | 92 | 2 | 56364 | 46 | 56628 | 56628 | 56706 | 200 | 3.998 |
| 228053_s_at | *TOMM5* | 48149 | 48049 | 155 | 47698 | 102 | 16 | 47916 | 47914 | 48029 | 276 | 3.997 |
| 223249_at | *CLDN12* | 43830 | 43726 | 102 | 0 | 43414 | 24 | 43682 | 43682 | 43726 | 52 | 3.995 |
| 225036_at | *TOMM5* | 60274 | 60058 | 236 | 59444 | 208 | 34 | 59782 | 59770 | 60028 | 112 | 3.988 |
| 214597_at | *SSTR2* | 311 | 311 | 307 | 0 | 4 | 0 | 311 | 311 | 311 | 13 | 3.055 |
| 1561266_at | *---* | 314 | 314 | 310 | 2 | 2 | 0 | 314 | 314 | 314 | 13 | 3.054 |
| 1556987_s_at | *OR2H1* | 343 | 343 | 338 | 0 | 5 | 0 | 343 | 343 | 343 | 13 | 3.052 |
| 237286_at | *INTS9* | 305 | 305 | 299 | 2 | 4 | 0 | 305 | 305 | 305 | 10 | 3.052 |
