# Supplementary material for: Random forest of perfect trees: concept, performance, applications and perspectives
Source: Bioinformatics. 2021 Feb 1;37(15):2165–74. doi: 10.1093/bioinformatics/btab074 (PMC8352507; doi:10.1093/bioinformatics/btab074)
Supplement: btab074_Supplementary_Data [file btab074_supplementary_data.zip › Supplementary data 3 revision2.docx]

Supplementary data 3 : Optimal number of probes according to the SVM-RFe procedure





| Number of probes | CA | MCC |
| --- | --- | --- |
| 2 | 0.759 | 0.492 |
| 3 | 0.782 | 0.534 |
| 4 | 0.789 | 0.541 |
| 5 | 0.817 | 0.614 |
| 6 | 0.833 | 0.660 |
| 7 | 0.848 | 0.693 |
| 8 | 0.860 | 0.720 |
| 9 | 0.873 | 0.747 |
| 10 | 0.873 | 0.747 |
| 11 | 0.879 | 0.751 |
| 12 | 0.876 | 0.750 |
| 13 | 0.876 | 0.752 |
| 14 | 0.881 | 0.760 |
| 15 | 0.886 | 0.772 |
| 16 | 0.885 | 0.765 |
| 17 | 0.881 | 0.759 |
| 18 | 0.884 | 0.767 |
| 54675 | 0.892 | 0.811 |
