# Supplementary material for: Random forest of perfect trees: concept, performance, applications and perspectives
Source: Bioinformatics. 2021 Feb 1;37(15):2165–74. doi: 10.1093/bioinformatics/btab074 (PMC8352507; doi:10.1093/bioinformatics/btab074)
Supplement: btab074_Supplementary_Data [file btab074_supplementary_data.zip › Supplementary data 4 revision2.docx]

Supplementary data 4 : Comparison of the probes selection according to the SVM-RFE, RFPT Hierarchical method and RFPT_NICs Score method.

| Model | Model Selection | MCC | CA |
| --- | --- | --- | --- |
| SVM | SVM-RFE | 0.926+/-0.11 | 0.966+/-0.05 |
| SVM | RFPT Hierarchical Ranking | 0.639+/-0.16 | 0.832+/-0.08 |
| SVM | RFPT NICs Score | 0.934+/-0.11 | 0.970+/-0.05 |
| LR | SVM-RFE | 0.857+/-0.19 | 0.930+/-0.10 |
| LR | RFPT Hierarchical Ranking | 0.919+/-0.16 | 0.960+/-0.07 |
| LR | RFPT NICs Score | 0.792+/-0.27 | 0.896+/-0.13 |
| RF | SVM-RFE | 1+/-0 | 1+/-0 |
| RF | RFPT Hierarchical Ranking | 1+/-0 | 1+/-0 |
| RF | RFPT NICs Score | 1+/-0 | 1+/-0 |

The MCC and CA metrics across the 3 models were consistently high, with a slight advantage for the RFPT_NICs Score method.
