# Supplementary material for: Random forest of perfect trees: concept, performance, applications and perspectives
Source: Bioinformatics. 2021 Feb 1;37(15):2165–74. doi: 10.1093/bioinformatics/btab074 (PMC8352507; doi:10.1093/bioinformatics/btab074)
Supplement: btab074_Supplementary_Data [file btab074_supplementary_data.zip › Supplementary data 2 revision2.doc]

Supplementary data 2 : Breast Cancer Wisconsin dataset analysed by our RPTF model, comparison with the LR model

The analysis involving all 569 complete observations. For each tree, 15 features were selected at random. In total, 27 276 trees were built, leading to 14 368 PTs (52.68%).

Comparisons between the features selected by Breiman’s random forest and those selected by our RPTF model

Logistic regression models were compared using the top 10 of the most important features according to each information criterion (Gini index or Nguyen’s information criteria). These results showed a better selection performance of the RFPT model than the RF model based on the AIC.
